# Annual Address of the President of the Illinois State Medical Society

**Published:** 1856-12

**Authors:** N. S. Davis


### SELECTIONS.

*Annual Address of the President of the Illinois State Medical Society, read at the Annual Meeting in Vandalia, June 4th,* 1856. By N. S. DAVIS, M. D.

*Gentlemen of the Society* :—By no means the least difficult part of my task, in preparing for the present occasion, has been the selection of a proper subject for our mutual consideration. Those miscellaneous topics relating to the education and ethics of our profession have constituted the theme of so many introductory and anniversary discourses, that they have lost all their novelty, if not their interest. On the other hand, I fear the full consideration of any particular disease would involve details alike tedious and inappropriate. A brief review of the present position of medical science, the tendencies of prevailing medical opinions, the special topics needing further investigation, and the best methods of conducting inquiries in relation thereto, would each and all constitute the subject of a discourse highly appropriate for an occasion like the present; but the proper consideration of such a theme requires far more time than has been at my command. I shall, therefore, relinquish these, and humbly invite your attention to the discussion of a single question, but one which, I trust, will not be devoid of either scientific interest or practical value. The question to which I refer, may be stated as follows, viz: What influence are Alcoholic Liquids capable of exerting, either in preventing or curing tubercular disease of the lungs?

Whether we regard tubercular phthisis in its character as the opprobrium of the healing art, or as the universal scourge of Christendom, giving rise, annually, to more than one-tenth of the whole number of deaths that occur, it is. in either aspect, a subject worthy of the most rigid and persevering investigation. On the other hand, the relations which alcoholic liquors bear to the great social and moral interests of human society, invest every proposed application of them, even in the treatment of disease, with a peculiar interest and importance. There is, perhaps, no disease in the whole catalogue which better illustrates the mutations of medical opinions and the influence of popular theories on medical practice than tubercular phthisis. At one time, it is regarded as the direct result of the sudden atmospheric changes which occur in all countries north of the tropics; and, to counteract its progress, the patients were carefully confined in heated rooms, and treated with tar, napthar and various medicated vapors. Next came the doctrine, that tubercula, disease was simply the result of repeated congestions, and a slow inflammation of the pulmonary tissue; and the exhaustion of the patients was rendered more rapid by repeated small bleedings, blisters, tartar emetic sores, setons, antimonials, low diet, and sometimes mercurials; and, when they could bear these no longer, a change of climate relieved the Doctor, if not the patient.

But, within the last twenty years, as you are well aware, all this has been reversed. There are very few, at the present time, who regard tubercular deposits in the lungs as the product either of inflammation or of the congestions consequent upon ordinary atmospheric vicissitudes. On the contrary, they are almost universally attributed to some defect in the processes of assimilation and nutrition, arising either from hereditary predisposition, or from causes which depress the vital forces and render the organic actions defective. And even the few who still claim the agency of congestion or inflammatiom in the generation of tubercular deposits, attribute these primary morbid conditions to debility or depression of the vital forces. Thus, Dr. Henry Goadby, of Detroit, in a recent article on Tuberculosis, says: "It invariably happens, in patients having a tendency to this disease, that the *vital powers are depressed* and the circulation feeble—the heart, in fact, has not sufficient power to propel the blood throughout the capillary phexuses which eminently distinguish the tissues the subjects of this disease."

Again, Dr. Theophilus Thompson, physician to the Hospital for Consumption and Diseases of the Chest, in London, when speaking of the *modus operandi* of the causes that favor the development of tubercles, says: "The unfavorable influences noticed in this lecture may be regarded as producing their effect, first, by *deteriorating the blood;* secondly, by occasioning congestion in the lungs." No sooner had this doctrine of debility, degeneration of the blood, and defective nutrition received the assent of the profession, than the old custom of resorting to warm air, depletive, and counter-irritation was abandoned, and various measures adopted, all having for their object the improvement of the strength and nutrition of the patients. At first cautious exercise in the open air by riding, a more nutritious diet, alterant tonics, such as iodine with bitter infusions, and anodynes to allay cough and irritability, were resorted to. But, seeing the almost uniform deficiency of fatty tissue in the consumptive, even in the incipient stages of the disease, and the extreme emaciation of the later periods, it was natural to infer that the radical defect in the nutritive processes was owing either to a deficient assimilation of fatty matter, or to an inordinate action of oxygen on the carbonaceous matter of the blood and tissues. Under the guidance of either of these ideas, with the medical mind thoroughly imbued with the chemico-physiological doctrines of Liebig in relation to the separate and specific offices filled by the two great classes of aliment, namely, the *nitrogenous* and non-nitrogenous or hydro-carbonaceous, it was to have been expected that the chief agents for counteracting the development and progress of tubercular disease, would be sought for in the latter class. Hence, we soon had added to the list of remedial agents, not only an ordinarily nourishing diet and free exercise in the open air, but also the use of cod-liver oil, cocoa-nut oil, fusil oil, and fat meats *ad libitum.* With all those patients whose digestive organs were capable of receiving and assimilating these oleaginous substances, they proved more or less beneficial, by lessening the rapidity of waste or emaciation and sustaining the strength; and, hence, the cod-liver oil quickly obtained a popularity and extent of use rarely equalled by any other remedy. Unfortunately, however, a large proportion of patients were soon nauseated by its use, and the profession were compelled to seek from the class of hydro-carbonaceous substances some substitute more palatable and yet calculated to fulfil the theoretical idea of neutralizing the action of oxygen on the tissues. For this purpose the various liquors, having alcohol as their active constituent, being at once palatable and ranked by Liebig and others as the most efficient and active of the whole class of hydro-carbonaceous substances, were readily chosen. At first, they were given cautiously and chiefly as vehicles to cover the taste and lessen the nauseous qualities of the oils; but, during the last year or two, they have been rapidly assuming a predominance and popularity which should at once challenge the closest scrutiny of the profession. So true is this, that free, nay severe, exercise in the open air, plenty of beef-steak, and wine and brandy *ad libitum*, constitute the popular prescription for the consumption of the present day, and that too by medical men of high reputation.

That I may not be accused of exaggeration, I will quote part of an article from the editor of the *Buffalo Medical and Surgical Journal*, as follows:

"The contrast between the two ideas of treatment is a marked one. The venesection, the emetic, the blister, the iodine inhalations, the careful protection from air and from exertion, the abstinence from animal food and from stimulating drinks, incident to our former ideas, have given place to active exercise, to fat meats, and hearty diet, to vinous and alcoholic stimulants, and to what would once have been deemed reckless exposure to vicissitudes of weather.

"'I've been bleeding from the lungs since I saw you!' said a medical friend as he entered our office, and in reply to our inquiry as to what he had done for it, he answered coolly, *drinking whisky punch!* He followed up this pleasant remedy in moderation, and, so far as we know, has had no return of his attack."

After alluding to one or two other cases in the same general terms, the writer adds:

"We could multiply cases. Even in those far advanced in the disease, we have *never failed* to witness more or less improvement as the result of this regimen. If it is the most promising of cure to the curable patient, it is also the most conducive to comfort in the incurable. We do not intend to discuss the pathology of the disease, or the rationale of the treatment in this hasty sketch; we wish only to declare our full conviction of the merits of the tonic treatment."

The article from which we have just quoted, was first published in the April number of the *Buffalo Journal*, and it has already been either copied, or alluded to approvingly, by a large portion of the medical journals of this country. It is true that some of them qualify their approval of the remedy by a word of caution in regard to the moral effect of a general recommendation of alcoholic beverages in any disease. Thus, the May number of the *St. Louis Medical and Surgical Journal* contains the following:

"*Alcoholic Treatment of Tuberculosis:*—The able editor of the *Buffalo Medical Journal* bears testimony to the beneficial effect of alcoholic liquors in the treatment of phthisis. We fully agree with him that the most successful treatment will be found to be the alcoholic, combined with nourishing diet, including cod-liver oil and the like, and constant, and even violent exercise in the open air. But there is a serious moral objection to this treatment, which is worthy of consideration. *It does tend to make drunkards, and perpetuate the vice of intemperance.* The physician who cures his patient of consumption, only to precipitate him into a drunkard's grave, has surely done him anything but a favor. What we mean to say is this, that so dangerous a remedy as alcohol should be recommended with very great caution, even in cases in which it is clearly beneficial."

Having made the physiological effects of alcohol, on the various functions of the human system, a special subject of study and experiment for several years past, and seeing clearly the tendency of medical opinion in favor of its use in the treatment of tuberculosis, I have, during the last twelve months, made a careful record of all the observations which have come within my reach, in relation to its influence both as a prophylactic and a curative agent for this disease. There are two distinct, but equally legitimate, methods of determining the value of any remedial agent. The first is purely philosophical, or inductive, and requires, on the part of the physician, an accurate knowledge of the nature and tendencies of the disease, together with an equally reliable knowledge of the natural or physiological effects of the proposed remedy. With such knowledge to constitute his premises, he may very confidently deduce conclusions concerning the effects of the agent, when applied to the prevention or cure of the disease The second method is wholly empirical or experimental, consisting in the direct observation of the effects which follow the administration of any medicinal agent, without reference to any previous knowledge of the physiological relations of the agent. When both these methods of investigation are employed in relation to the same subject, and the results obtained mutually corroborate each other, the evidence or knowledge afforded is scarcely less certain than though it had been obtained by a mathematical demonstration. In further pursuing the question under discussion, I shall employ both the methods just named: the first briefly, but the latter more in detail.

In entering upon the first, two questions are presented for solution as the basis of all the subsequent steps, viz:

*First.* What are those conditions of the human system which constitute pulmonary consumption or a predisposition thereto; or, in other words, What is the true pathology of tuberculosis?

*Second.* What are the actual changes in the fluids and solids, or the properties and functions of the human system, produced by alcoholic liquors?

By a critical examination of the facts and opinions contained in our medical literature, we may reduce the answer to our first inquiry to the following propositions, viz: Tuberculosis essentially consists in the formation and deposit of a partially-organized matter, which affords, by chemical analysis, most of the proximate and ultimate constituents of the healthy structures, and, under the microscope, is seen to consist of cells, many of which are imperfect, nuclei, and an amorphous granular substance; all of which seems to possess a strong tendency to further degeneration and the ultimate formation of pus. The conditions favorable to the development of the tubercular matter, are, either such a defect, hereditary or acquired, in the primary organization of the individual, that the processes of assimilation and nutrition are incapable of elaborating all the primary cell formations in that perfection necessary to fit them for constituting healthy structures; or a similar defect in the process of disintegration, by which the cells and fibrin, once constituting healthy tissue, are returned into the blood so imperfectly disorganized as to be incapable of excretion, and hence accumulate until they find a lodgement in some of the tissues. With either of these pre-dispositions, or rather defects, the place where and the time when the deposit will commence, and the rapidity of its increase, will depend much on accompanying circumstances. If the primary defect is in the assimilative process, and it be strongly manifested in early life, the more vascular structures, directly connected with that process, will become the local seat of disease, and we shall have the ordinary scrofulous enlargement of the lymphatic and mesenteric glands. On the other hand, if the defect be slight, or chiefly in the process of disintegration, and especially if it is not developed until the middle or later periods of life, the deposit will be by far the most frequent in the lungs; owing, no doubt, to their extreme vascularity—their liability to frequent congestions—and their being the seat of one of the most important excretory functions of the whole system. Such is a concise statement of the most enlightened views at present entertained in regard to the nature and development of tubercular disease, whether located in the lungs or elsewhere. So far as they are correct, it is evident that whatever tends to invigorate the system, improve the tone of the organized structures, and render the process of assimilation more perfect, on the one hand; and, on the other, whatever tends to aid the free oxygen in disintegrating and more perfectly excreting the matter of the tissues as they become effete or useless, tends directly to prevent or arrest the process of tuberculosis.

Hence, whatever may be the diversity of views in regard to other matters, all at the present day agree that pure air, free muscular exercise, a nutritious diet, and a cheerful frame of mind, are the best prophylactics, if not curative agents, in phthisis; while confined and impure air, insufficient or indigestible food, sedentary habits, and depressing mental emotions, as certainly favor the development of the disease and hasten its fatal termination.

Our next step is to inquire concerning the effects of alcohol upon the blood, and, through it, upon the assimilative and depurative processes of the system, that we may see clearly its therapeutic relations, not only to phthisis, but to all other diseases.

There may be those who will regard a serious inquiry into the *modus operandi* of an agent, so long and so generally used as alcohol, entirely superfluous, it having long since been fixed, both in the popular and professional mind, as one of the most efficient stimulants and supporters of the temperature of the system which we possess. Whatever truth there may be in the old maxim, *Vox populi*, *Vox Dei*, when applied to politics, it requires but little research to show the entire fallacy of the *vox populi* when applied to questions of science or medical philosophy. If we drop those popular notions and theories which we have unconsciously imbibed with our education and intercourse with society, and subject alcohol to the same experimental tests as are required to determine the effects of other agents, we shall be much more likely to arrive at reliable results. Such experimental tests have been applied at various times, and by some of the most eminent investigators of the present day, and the results have been so uniform as to leave no doubt in regard to their correctness.

When alcohol is administered in moderate quantities, one of its first and most prominent effects, (that indeed which chiefly attracts the attention of the community,) is a peculiar exhilaration of the brain and nervous system, coupled with a weakness or unsteadiness in the actions of the voluntary muscles.

While in this state, however, other effects, of far more physiological importance, are taking place in the blood and the elementary functions of the system. The usual proportion of carbonic acid gas fails to be thrown off in the expired air, as shown by the experiments of Drs. Prout, Bouchardet, myself, and others.* Carbon, consequently, accumulates in the blood, diminishing the change from venous to arterial hue in the lungs, and directly diminishing all the organic and secretory actions of the system. As further and more demonstrative proof of the latter part of this assertion, it has been shown by recent experiments, that, when alcoholic drinks are taken even in moderate quantities, the aggregate amount of all the excretions from the body are less, in a given time, than when no alcohol is present in the system. Another proof of the same diminution of organic change, both nutritive and secretory, was developed by my own experiments, began in 1850, and repeated at various times since,† by which it was shown, that the diminished excretion of carbonic acid from the lungs, under the influence of alcoholic liquors, was accompanied and followed by a positive diminution of the temperature of the body. Again, when death has been produced, either in animals or men, by an overdose of alcohol, the blood is not only found of a dark, venous color in the arteries as well as veins, but the coagulability of its fibrin is also impaired. I shall not trespass on your patience to inquire here, whether these effects of alcohol on the blood, and the functions of respiration, secretion, and animal heat, are owing to the direct affinity of the carbon and hydrogen of the alcohol for the free oxygen of the blood, by which the latter is prevented from exerting its proper influence in the processes of nutrition, disintegration, and excretion; or whether they are owing to the direct antiseptic qualities of the alcohol, by which it tends to arrest all change in organic matter, whether living or dead; a brief statement of the effects alone being all that the limits and objects of this address will permit.

In answering the inquiry, what are the effects of alcohol on the human system, all my experiments and observations lead to the following conclusions, viz:

1st. The presence of alcohol in the blood produces a temporary exhilaration or excitement of the nerve structures.

2d. It diminishes the excretion of carbonic acid from the lungs; diminishes the change of color from venous to arterial blood; and diminishes generally the organic changes in the system.

3d. It depresses the temperature of the body, and lessens the tone of the muscular structures.

If these conclusions are correct, (and it would afford me great pleasure to give the facts and experiments in detail from which they are deduced, were it possible within the limits proper for an address,) every member can see clearly the relations which they bear to the acknowledged conditions accompanying the development and progress of phthisis.

---

* See Thompson's Annals of Philosophy, Vol. II.; also, Carpenter's Human Physiology, p. 306.

† See *North-Western Med. and Surg. Journal,* 1851; also Address on Alcoholic Liquors, &c., Feb., 1855.

It is easy to perceive that the presence of alcoholic liquids in the system can neither promote healthy nutrition, nor facilitate the processes of disintegration and excretion, and, therefore, that there is no rational or inductive indication for their use, either for the prevention or cure of consumption. The idea advanced by Dr. Goadby, in the article already referred to, namely, that the immediate cause of pulmonary tubercles is the inability of the heart to circulate the blood freely through the intricate plexuses of the pulmonary capillaries, affords still less indication for the use of brandy or any other alcoholic liquid. For there is no fact in physiology better established than, that whatever lessens the change from venous to arterial blood in the lungs, or tends to accumulate effete carbon in the blood, directly lessens the facility with which that fluid flows through the capillaries of those organs. And yet it has been an hundred times demonstrated by direct experiment, that alcohol, whether in the form of distilled or fermented liquors, produces speedily, and in a marked degree, precisely this effect on the blood. So marked indeed is this effect, that, in some of my experiments, the air exhaled from the lungs one hour after three or four ounces of brandy had been taken into the stomach, contained more than twenty-five per cent. less than the normal proportion of carbonic acid gas. And whoever will observe the purple lips, the bloated and leaden face, the slow and almost stertorous breathing of full intoxication, will see abundant proof of the impeded circulation through the pulmonary capillaries. Still, Dr. Goadby, and many others recommend the use of brandy and wine "to increase the *energy* and *power*" of the heart, and *invigorate* the system. We fear that they, like the multitude of non-professional men, have mistaken the exhilarating effects of these fluids on the brain and mind for a true increase of organic vigor and energy. Certain it is, that they have not furnished us with a single experimental or demonstrative fact in proof of their position. Some of my audience may be ready to ask, if I would deny the almost universal sentiment of mankind, by claiming the broad ground that alcoholic liquors are neither tonic, invigorating, or supporting to the human system? I answer, unhesitatingly, Yes! and base my answer not merely on the deductions of the most careful and varied experimental researches, but ask, in return, What there is in the tottering and unsteady step, the impaired digestion, the frequent functional derangements of the kidneys, exhibited in a greater or less degree by all habitual drinkers, which is indicative of increased physical strength, vigor, or power of endurance? But I must hasten to the last department of my task, in which I shall confine myself exclusively to the results of clinical experience.

About one year since, my attention was directed to this branch of the subject, and I searched diligently the pages of our medical literature for some record of observed facts in relation to the use of alcoholic liquids in the treatment of tubercular disease; but I sought in vain. There were many expressions of *opinion* in the same general phraseology as that already quoted from the *Buffalo Journal.* It was undoubtedly true, that the editor's friend had "been bleeding at the lungs"—that he had drank *whiskey punch*—and that he had no return of the attack, so far as he (the editor) knew. But, of what possible value is such a statement? It neither informs us when the bleeding occurred, how much punch he drank, *how long* he had remained exempt from the bleeding, or what else he had done besides drinking whiskey punch during the same time. It is only a few weeks since a young man came to me with extensive tubercular deposits in his lungs. He had never had any hemorrhage from the lungs, but his physician advised him to go South, live on hearty food, and drink pretty freely of brandy every day. He did so, and in less than six weeks he was taken with copious bleeding from the lungs. Now, if I should gravely set forth this case, as proof that brandy produced hemorrhage from the lungs, it would be precisely of the same value as the statement about the *whiskey punch.* An eminent teacher of surgery is in the habit of relating the case of a young man whom he supposed to be far advanced in consumption, but who went into the country, exercised freely, almost recklessly, in riding, hunting, fishing, &c.; lived on beef-steak and brandy, and returned home in apparent good health. But a writer in one of our south-western journals, relates another case equally advanced in disease, who also went into the country, endured the same reckless exposure, lived on nutritious food, and recovered equally good health, but without having used a drop of brandy or any other alcoholic liquid. I mention these merely as examples of what constitutes far too great a share of the practical part of our medical literature. *Post hoc, ergo propter hoc,* furnishes the only basis for far too large a proportion of our practical conclusions.

During the past twelve months, I have kept a careful record of all the well-marked cases of tubercular consumption which have come under my own observation. I have taken special care to ascertain both the present and preceding habits in regard to the use of alcoholic drinks in each individual case. The whole number of cases, so observed and recorded, is 37; of whom 10 were natives of the United States, 24 of Ireland, 1 of England, 1 of France, and 1 of Germany. Of the whole number, 26 were males and 11 females. Of the whole number only 6 were tee-totalers, or such as wholly abstain from the use of intoxicating liquors. Of the remaining 31, six drank alcoholic liquors only occasionally or at irregular intervals; while the remaining 25 drank either distilled or fermented liquors almost every day, until pulmonary disease had made such progress that they were compelled to desist, either from want of means to buy it with, or from its positively aggravating the disease under which they were laboring; and six of the number had been, one or more years, what the world calls *habitual drunkards.* If we had taken the utmost care to select cases for the purpose of demonstrating experimentally, whether alcohol possessed any power to control the progress of tubercular development, by its continued use through a series of years, we could not have made the experiments more satisfactory than some of the cases embraced in this record. We select the two following in relation to the use of beer:

*Case* 1—Mrs. D——, an Irishwoman, aged 21 years; has been in America two years; has been married eighteen months, and has one child, six months old. For two years before she came to America, she had been employed as a servant-girl in an English family; had been supplied with nutritious food, and a liberal allowance of beer, which she drank regularly and freely every day during the whole time.

After her arrival in this country, she continued to drink beer and occasionally other liquors, but neither so much nor so regularly as before. She began to have some cough before she sailed for this country, which has steadily increased, with all the accompanying symptoms of phthisis, until the present time. At present she is extremely emaciated, pulse rapid and feeble, exhausting night-sweats, copious purulent expectoration, with all the physical signs of extensive tubercular cavities in the upper lobe of both lungs. She says none of her immediate family are, or have been, consumptive, but she thinks one uncle died with that disease. About three weeks after the foregoing record was made the patient died.

*Case* 2—Mr. B.——, a native of Ireland, aged 22 years; is clerk in a liquor store, or, in other words, a "bar-tender;" has been kept much within doors for more than two years; no proof of very direct hereditary pre-disposition, though some of his relatives have died from phthisis. He says he has drank regularly every day from three to six glasses of beer, during the whole of the last two years, and occasionally a glass of other liquor. He began to cough about six months since, and has continued to do so, accompanied by steadily-increasing emaciation, and all the ordinary symptoms of consumption.

He has now purulent expectoration mixed with tubercular matter, pulse from 90 to 110 per minute, frequent night sweats, and impaired digestion. The chest is flattened, especially beneath the left clavicle; dull on percussion over the same region, the respiratory murmur prolonged and cavernous, accompanied by a crackling and sub-crepitant ronchus, while the voice furnishes distinct broncophony. There can be no doubt but the upper lobes of the lungs are filled with a copious tubercular deposit, at present in the second stage of advancement, or that of rapid softening.

In these two cases it will be observed that the use of beer was commenced, in one, eighteen months, and the other, two years before any active symptoms of tuberculosis were known to exist. And, though the drink was taken regularly, and for the most part at meal-time, it seemed to exert not even a retarding influence over the development of the disease.

In regard to the use of distilled spirits, some of the cases present still more striking experiments, but we will quote from our memorandum-book only the two following:

*Case* 3—Mr. M——, a native of Ireland, but a resident in this country for fifteen years, aged 35 years; of medium size, dark hair, and apparently a well-developed frame.

Says he does not know of any cases of consumption in his own family, or among his more direct ancestors. He has been a country hotel-keeper during the last twelve or fifteen years, during all of which time, he has drunk more or less alcoholic drinks, generally distilled spirits, almost every day, but not to the extent of producing intoxication. His wife died during the summer of 1854, and, for the succeeding eight or nine months, he drank liquor more freely, and continued to do so until the following spring, when he first began to be troubled with cough and symptoms of pulmonary disease. After the smyptoms of disease had made considerable progress, he says he was compelled to forego the further use of stimulating drinks, on account of their "making him worse."

Now, (Dec. 1855,) he is much emaciated, has copious and exhausting night-sweats, lips pale and thin, cheeks sunken, pulse 80 per minute in the morning and very feeble, in the evening, 120 per minute; appetite much impaired, bowels regular, cough frequent and cavernous, expectoration copious and purulent, with a distressing sense of suffocation at times. There is much depression of the infra-clavicular regions, but more of the right than the left. Over the same regions there is dulness on percussion, the respiration cavernous with gurgling rhoncus, and the voice affording plain pectorilogy—in other words, there were large tubercular cavities in the upper lobes of both lungs. This patient died in about ten days after the foregoing record was made.

*Case* 4—Michael ——, aged 38 years; native of Ireland, but lived in this country several years; by occupation, a laborer; accustomed to constant out-door work, and the use of hearty food; does not know that there is any hereditary tendency in his family to tubercular disease. Says he has drank all kinds of alcoholic liquors freely, but especially whisky, almost daily for the last fifteen years, and continued to do so, until his present disease had made such progress that he was compelled to desist. He commenced coughing about eight months since, but continued to work pretty steadily until the last two months. At present he presents every symptom and sign, rational and physical, of extensive tubercular disease of the lungs in the last stage of its advancement, as plainly as those detailed in Case 3. He died in the Mercy Hospital, about one week after he came under my observation.

Several other cases might be selected from the above list, strikingly illustrating the inefficiency of all the varieties of alcoholic drinks, either for preventing or curing consumption; but I have given enough to show that, whether they are viewed in the aggregate or in detail, they afford no evidence in favor of the therapeutic value of alcoholic drinks in the treatment of tuberculosis. If you ask how I account for the prevalent, popular and professional opinions in their favor, I answer, in three ways.

*First*, these opinions are based, with many, exclusively on the delusive idea, still very prevalent, that these liquors are actually tonic or invigorating to the human system under ordinary circumstances. They say, consumption is a disease of debility—alcoholic liquors are tonics—therefore, alcoholic liquors are beneficial in that disease. Like a recent writer in the *Westminster Review*, they reason *logically*. To prove that alcohol was food, he said "food is *force*"—"alcohol is force"—"therefore, alcohol is food."

Both employ syllogisms, but both forget to prove their premises; and both forget that we might, with just as good logic, say that food is force—steam is force—therefore, steam is food.

*Second*. The influence of alcohol in exhilarting the brain, in many cases, lessens, for a time, the extreme sensitiveness which accompanies some cases of consumption, renders the patient more tranquil, and thereby produces an apparent temporary advantage. Another influence of alcohol which I have described, namely, its tendency to diminish the organic actions and excretory functions of the system, enables it in some cases of phthisis, characterized by rapid emaciation, to check that process, and, in some instances, to give an apparent increase of nutrition by retaining more of the fatty matter in the system. But, in every case that has come under my observation, this apparent benefit has been only temporary, the apparent increase of nutrition unhealthy, and the patients in the end sink more rapidly than ordinary cases. In one instance of this kind, a *post-mortem* revealed extensive uncicatrized cavities in the lungs, extensive fatty deposits in the liver, and slight fatty degeneration of the muscular structure of the heart. Some have claimed that alcohol, by diminishing the process of disintegration and waste, performed an office equivalent to the taking of more food. If this were true, opium would be much more efficient food than alcohol ; and we should only require some agent capable of arresting disintegration altogether, to enable us to live perpetually without the inconvenience of paying board-bills.

*Third.* The greatest cause of error, in estimating the effects of alcohol on the consumptive, is the universal custom of recommending, along with the alcoholic drinks, a thorough change of habits, active exercise, nutritious food, and often a change of climate ; and, then, making no distinction between the effects of the alcohol and the accompanying circumstances. All know, that, in many cases of chronic disease, if we give the patient bread-pills, and exact the proper exercise, diet, &c., they get well. They, of course, are willing to swear that the pills cured them: and, I much fear, that, in reference to alcoholic drinks, many physicians not only deceive themselves but their patients also. Two years since, I knew a gentleman of our city who found himself, in the autumn, reduced to a very feeble condition in advanced phthisis. He had been closely shut up by Homœopaths for two months; had hectic, night-sweats, purulent expectoration, and well-formed cavities in one lung. Being called upon for advice, I told him he had an incurable disease, but if he wished to prolong life, he must ride out every day, take plain, easily-digestible, and nutritious food, and, for medicine, cod-liver oil and the cold infusion of wild cherry bark. He followed my advice. At first he had to be carried to his carriage ; but he soon gained sufficient strength to get in and out alone. He continued to ride through all the cold of winter, did a good business in buying pork for packing, but sunk and died during the first warm weather of spring. A *post-mortem* revealed simply extensive tubercular deposits and cavities in both lungs.

Now, if I had prescribed for that patient, a glass of brandy or wine every day, and it had not positively prevented the favorable results, the latter would, most certainly, have been ascribed in a great measure to it. But he took not a single glass.

The great leading object of this address, is, to arrest the attention of the profession, and to entreat its members to adopt more rigid and careful modes of investigation ; at least to avoid giving currency to those loose, general statements which can only deceive the sick, and sometimes give encouragement to habits which may plunge thousands of the well into irretrievable ruin. If I accomplish this, I shall feel amply repaid for all the labor bestowed.

